# Differential relationships of gait velocity mean and variability with cognitive performance in preclinical older adults

**DOI:** 10.1002/alz70856_106219

**Published:** 2026-01-07

**Authors:** Wajiha Ahmed, Allal Boutajangout, Jon Links, Natasha De La Cruz, Brianna Vega, Alok Vedvyas, Ludovic Debure, Gaurav Vedvyas, Sakina Ouedraogo Tall, Omonigho M Bubu, Joshua Chodosh, Ricardo S. Osorio, Karyn Marsh, Thomas Wisniewski, Arjun V. Masurkar

**Affiliations:** ^1^ NYU Grossman School of Medicine, New York, NY, USA; ^2^ NYU Alzheimer's Disease Research Center, New York, NY, USA; ^3^ Center for Cognitive Neurology, New York University Langone Health, New York, NY, USA; ^4^ Alzheimer's Disease Research Center, New York University Langone Health, New York, NY, USA; ^5^ New York University Grossman School of Medicine, New York, NY, USA

## Abstract

**Background:**

Gait decline may serve as a critical and early indicator for cognitive impairment, yet these associations have primarily been examined qualitatively, with a focus only on means, and primarily restricted to cohorts of impaired individuals. We have previously identified differential relationships of quantitatively measured gait velocity mean and intra‐individual variability (IIV) with AD‐related plasma biomarkers in preclinical older adults. Here we aimed to explore their relationships to objective cognitive performance and examine if quantitatively measured mean and IIV of gait velocity exhibit distinct relationships.

**Method:**

This cross‐sectional study included 258 cognitively normal older adults (74.8% White, 22.1% Black/ African American, and 2.3% Asian) from the NYU Alzheimer's Disease Research Center cohort who underwent quantitative gait analysis via the GAITRite electronic walkway. Mean and IIV of gait velocity were assessed over eight laps. Cognition was assessed using the Montreal Cognitive Assessment (MoCA), Mini‐Mental State Examination (MMSE), and other tests included in the National Alzheimer's Coordinating Center's Uniform Data Set 3.0. Depressive symptoms were assessed via the Geriatric Depression Scale (GDS). The analysis utilized multiple linear regression models, adjusted for age, sex, education, and GDS.

**Result:**

Gait velocity IIV was negatively correlated with MMSE (*p* = 0.02). Mean gait velocity positively correlated with the MoCA (*p* = 0.01), number span backward (*p* = 0.03), category fluency (animals and vegetables) (*p* = 0.02), verbal fluency (*p* = 0.05). Additional trends suggested that lower cognitive scores were associated with increased IIV and lower mean gait velocity value.

**Conclusion:**

Our results demonstrate that IIV and means of gait velocity show differential relationships with cognitive tests in preclinical individuals. This suggests that gait velocity IIV may have a unique predictive role in the preclinical state distinct from gait velocity mean. Further longitudinal studies are warranted to address the correlations of mean versus IIV of gait velocity with future objective cognitive decline.

